# Hospital hand hygiene after COVID-19: has the pandemic heightened healthcare workers’ awareness? Giacomo Pietro Vigezzi

**DOI:** 10.1093/eurpub/ckac129.182

**Published:** 2022-10-25

**Authors:** M Zeduri, AC Sgueglia, GP Vigezzi, P Ferrara, M Lanave, R Galvi, S Abela, V Novelli, A Muzzi, A Odone

**Affiliations:** 1 Department of Public Health, Università degli Studi di Pavia, Pavia, Italy; 2 Medical Direction, Fondazione IRCCS Policlinico San Matteo, Pavia, Italy

## Abstract

**Background:**

Hand hygiene (HH) is the leading measure for preventing the transmission of healthcare-associated infections (HAI), and a cornerstone to prevent COVID-19 spread. Aim of the research was the assessment of HCWs’ adherence to the application of WHO optimal practices, with the goal to promote a culture of safety and quality infection prevention and control (IPC) activities.

**Methods:**

Fondazione IRCCS Policlinico San Matteo, Pavia, Italy, implemented a HH monitoring plan in which HCWs’ adherence to HH procedures is evaluated using WHO guidelines, technical manual and observation form. Direct field observations took place in March and April 2022 by trained personnel. Process index was HH adherence, stratified by profession, opportunity and unit, which has been visited at least twice.

**Results:**

Overall, 302 HCWs were observed from 18 hospital units (105 physicians, 108 nurses, 84 healthcare assistants and 5 students). Out of 1382 opportunities, global adherence was 52% with 190 handwashing and 598 hand rubbing. The indication with the highest adherence was “after body fluid exposure risk” (76%), whereas the lowest were “after touching the patient's setting” (40%) and “before touching a patient” (43%). Adherence was higher in specialistic surgeries and haematology units, while the worst performances were reported in general medicine ward (29%). Physicians’ and nurses’ adherence was respectively 45% and 61%. Audits occasionally revealed non-conformities in glove use (i.e., unnecessary use, not changed between patients, hand rubbing on gloves).

**Conclusions:**

These preliminary findings could be directly linked to habits acquired during the pandemic, when HW tended to consider COVID-19 patients as a unique block to shield themselves from infections, rather than safeguarding individual patient units. HH awareness could have changed in the wake of COVID-19 pandemic and our study described how HCWs’ adherence to optimal practices needs specific initiatives to promote correct HH.

**Key messages:**

• The COVID‐19 pandemic reinforced the importance of handwashing and IPC, showing the key role of the HCWs’ adherence to hand hygiene (HH) procedures.

• HH audits play a leading part in clinical governance and IPC, aiming at enhancing the quality of care and patient safety, particularly to strengthen health system resilience in post-COVID era.

